# 2,9,16,23-Tetra­kis(1-methyl­eth­yl)-5,6,11,12,13,14,19,20,25,26,27,28-dodecadehydro­tetra­benzo[*a*,*e*,*k*,*o*]cyclo­eicosene^1^

**DOI:** 10.1107/S1600536811048604

**Published:** 2011-11-30

**Authors:** Theshini Perera, Frank R. Fronczek, Steven F. Watkins

**Affiliations:** a Department of Chemistry, Louisiana State University, Baton Rouge, LA 70803-1804, USA

## Abstract

The title compound, C_48_H_40_, is a tetra­isopropyl-substituted polyannulenoenyne. The unsubstituted polyannulenoenyne, C_36_H_16_ (CSD: RICVEE; CAS: 186494-87-1), has quasi-*D*_2_ (222) symmetry, as determined by least-squares fit (excluding H atoms) to a model optimized in *D*_2_ symmetry by mol­ecular mechanics (r.m.s. deviation = 0.239 Å). The least-squares fits of 36 common C atoms of the title compound (at 90 K) to the parent (at 295 K) and to the optimized model show r.m.s. deviations of 0.419 and 0.426 Å, respectively.

## Related literature

For a description of the Cambridge Structural Database, see: Allen (2002 ▶). For the synthesis and a related structure, see: Boese *et al.* (1997 ▶). For mol­ecular mechanics software, see: Cambridgesoft (2010 ▶).
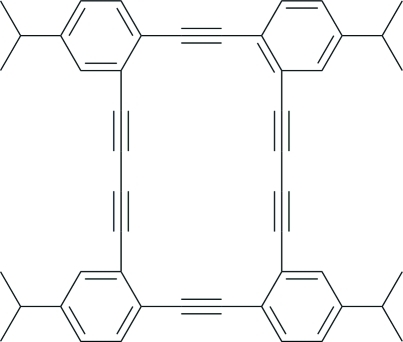


## Experimental

### 

#### Crystal data

C_48_H_40_
*M* *_r_* = 616.8
Monoclinic, 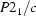

*a* = 18.0007 (4) Å
*b* = 12.5083 (3) Å
*c* = 16.0674 (4) Å
β = 91.004 (1)°
*V* = 3617.15 (15) Å^3^
*Z* = 4
Mo *K*α radiation
μ = 0.06 mm^−1^
*T* = 90 K
0.45 × 0.30 × 0.30 mm

#### Data collection

Nonius KappaCCD diffractometer
Absorption correction: multi-scan (*SCALEPACK*; Otwinowski & Minor 1997 ▶) *T*_min_ = 0.972, *T*_max_ = 0.981
13998 measured reflections
8186 independent reflections
5354 reflections with *I* > 2σ(*I*)
*R*_int_ = 0.035

#### Refinement

*R*[*F* ^2^ > 2σ(*F* ^2^)] = 0.049
*wR*(*F* ^2^) = 0.127
*S* = 1.02
8186 reflections
442 parameters
H-atom parameters constrained
Δρ_max_ = 0.25 e Å^−3^
Δρ_min_ = −0.28 e Å^−3^

### 

Data collection: *COLLECT* (Nonius, 2000 ▶); cell refinement: *SCALEPACK* (Otwinowski & Minor, 1997 ▶); data reduction: *DENZO* (Otwinowski & Minor, 1997 ▶) and *SCALEPACK*; program(s) used to solve structure: *SHELXS97* (Sheldrick, 2008 ▶); program(s) used to refine structure: *SHELXL97* (Sheldrick, 2008 ▶); molecular graphics: *ORTEP-3 for Windows* (Farrugia, 1997 ▶); software used to prepare material for publication: *IDEAL* (Gould *et al.*, 1988 ▶) and *WinGX* (Farrugia, 1999 ▶).

## Supplementary Material

Crystal structure: contains datablock(s) global, I. DOI: 10.1107/S1600536811048604/kj2194sup1.cif

Structure factors: contains datablock(s) I. DOI: 10.1107/S1600536811048604/kj2194Isup2.hkl

Supplementary material file. DOI: 10.1107/S1600536811048604/kj2194Isup3.cml

Additional supplementary materials:  crystallographic information; 3D view; checkCIF report

## Figures and Tables

**Fig. 1.**
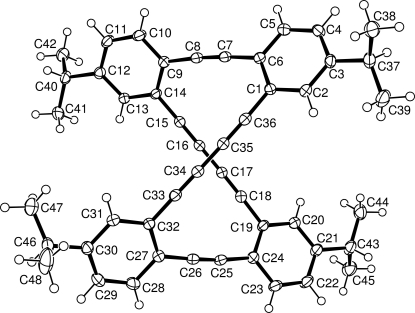
View of the title compound showing 50% probability displacement ellipsoids.

## supplementary crystallographic information

### Comment

The title compound, C_48_H_40_, is a tetraisopropyl-substituted polyannulenoenyne and its structure was determined at 90 K. The 295 K structure of the unsubstituted parent annulene, C_36_H_16_, was previously determined by Boese *et al.* (1997; CSD: RICVEE, Allen, 2002; CAS: 186494–87-1), and is of quasi-D_2_ (222) symmetry, as determined by least-squares fit (Gould *et al.*, 1988; δ_r.m.s._ = 0.239 Å) of all 36 common carbon atoms of the parent to a model of C_36_H_16_ optimized in D_2_ symmetry by molecular mechanics (Cambridgesoft, 2010). Reasonable agreements result from the same least-squares fit of the title compound (at 90 K) to the parent (δ_r.m.s._ =0.419 Å) and to the optimized model (δ_r.m.s._ = 0.426 Å).

The six C≡C triple bond distances are all experimentally equal, falling in the narrow range 1.203 (2) - 1.206 (2) Å, while the C–C bonds linking the triple bonds have length C16—C17 1.370 (2) and C34—C35 1.377 (2) Å. The acetylenic bridges are slightly bowed outward, with C–C≡C angles in the range 177.59 (18) - 179.17 (18)° for the butadiyne bridges and in the range 174.43 (17) - 176.80 (17)° for the acetylene bridges. Distances between the following bond centroids provide a measure of overall molecular dimensions: C16—C17 to C34—C35 = 3.341 (2) Å, C7 ≡C8 to C25 ≡C26 = 7.650 (2) Å.

### Experimental

The preparation of the title compound has been described by Boese *et al.* (1997). Crystals were grown by slow evaporation from dichloromethane and deuterochloroform.

### Refinement

All H atoms were placed in calculated positions guided by difference maps. The C—H bond distances were constrained to the range from 0.95 to 1.0 Å, depending on C atom type, and *U*_iso_= 1.2*U*_eq_ (1.5 for methyl groups), thereafter refined as riding. A torsional parameter was refined for each methyl group.

### Crystal data

**Table:**

C_48_H_40_	*F*(000) = 1312
*M**_r_* = 616.8	*D*_x_ = 1.133 Mg m^−^^3^
Monoclinic, *P*2_1_/*c*	Mo *K*α radiation, λ = 0.71073 Å
Hall symbol: -P 2ybc	Cell parameters from 7618 reflections
*a* = 18.0007 (4) Å	θ = 2.6–29.1°
*b* = 12.5083 (3) Å	µ = 0.06 mm^−^^1^
*c* = 16.0674 (4) Å	*T* = 90 K
β = 91.004 (1)°	Prism, golden yellow
*V* = 3617.15 (15) Å^3^	0.45 × 0.30 × 0.30 mm
*Z* = 4	

### Data collection

**Table:**

Nonius KappaCCD diffractometer	8186 independent reflections
Radiation source: sealed tube	5354 reflections with *I* > 2σ(*I*)
horizonally mounted graphite crystal	*R*_int_ = 0.035
Detector resolution: 9 pixels mm^-1^	θ_max_ = 28.9°, θ_min_ = 2.8°
CCD rotation images, thick slices scans	*h* = −23→23
Absorption correction: multi-scan (*SCALEPACK*; Otwinowski & Minor 1997)	*k* = −16→14
*T*_min_ = 0.972, *T*_max_ = 0.981	*l* = −19→20
13998 measured reflections	

### Refinement

**Table:**

Refinement on *F*^2^	Secondary atom site location: difference Fourier map
Least-squares matrix: full	Hydrogen site location: inferred from neighbouring sites
*R*[*F*^2^ > 2σ(*F*^2^)] = 0.049	H-atom parameters constrained
*wR*(*F*^2^) = 0.127	*w* = 1/[σ^2^(*F*_o_^2^) + (0.0543*P*)^2^ + 1.183*P*] where *P* = (*F*_o_^2^ + 2*F*_c_^2^)/3
*S* = 1.02	(Δ/σ)_max_ = 0.001
8186 reflections	Δρ_max_ = 0.25 e Å^−^^3^
442 parameters	Δρ_min_ = −0.28 e Å^−^^3^
0 restraints	Extinction correction: *SHELXL97* (Sheldrick, 2008), Fc^*^=kFc[1+0.001xFc^2^λ^3^/sin(2θ)]^-1/4^
0 constraints	Extinction coefficient: 0.0017 (4)
Primary atom site location: structure-invariant direct methods	

### Special details

**Table:**

Geometry. All e.s.d.'s (except the e.s.d. in the dihedral angle between two l.s. planes) are estimated using the full covariance matrix. The cell e.s.d.'s are taken into account individually in the estimation of e.s.d.'s in distances, angles and torsion angles; correlations between e.s.d.'s in cell parameters are only used when they are defined by crystal symmetry. An approximate (isotropic) treatment of cell e.s.d.'s is used for estimating e.s.d.'s involving l.s. planes.

### Fractional atomic coordinates and isotropic or equivalent isotropic displacement parameters (Å^2^)

**Table:**

	*x*	*y*	*z*	*U*_iso_*/*U*_eq_	
C1	0.34021 (9)	0.67572 (12)	−0.03559 (9)	0.0209 (3)	
C2	0.37458 (9)	0.65496 (13)	−0.11163 (9)	0.0230 (4)	
H2	0.3986	0.5882	−0.1196	0.028*	
C3	0.37425 (9)	0.72994 (13)	−0.17553 (9)	0.0239 (4)	
C4	0.33685 (9)	0.82602 (13)	−0.16283 (9)	0.0250 (4)	
H4	0.3348	0.8774	−0.2064	0.03*	
C5	0.30274 (9)	0.84840 (13)	−0.08857 (9)	0.0240 (4)	
H5	0.2781	0.9149	−0.0816	0.029*	
C6	0.30408 (9)	0.77426 (12)	−0.02347 (9)	0.0211 (3)	
C7	0.26840 (9)	0.79587 (12)	0.05395 (9)	0.0221 (3)	
C8	0.23760 (9)	0.80786 (12)	0.11918 (9)	0.0221 (3)	
C9	0.20160 (8)	0.81294 (12)	0.19792 (9)	0.0215 (3)	
C10	0.20544 (9)	0.90410 (13)	0.24814 (10)	0.0264 (4)	
H10	0.2289	0.9669	0.2282	0.032*	
C11	0.17525 (9)	0.90345 (13)	0.32691 (10)	0.0269 (4)	
H11	0.1793	0.9658	0.3604	0.032*	
C12	0.13916 (9)	0.81390 (13)	0.35838 (9)	0.0223 (3)	
C13	0.13314 (9)	0.72469 (12)	0.30736 (9)	0.0208 (3)	
H13	0.1073	0.6636	0.3267	0.025*	
C14	0.16417 (8)	0.72248 (12)	0.22813 (9)	0.0198 (3)	
C15	0.15945 (9)	0.62679 (12)	0.17971 (9)	0.0216 (3)	
C16	0.15709 (9)	0.54517 (12)	0.13983 (9)	0.0215 (3)	
C17	0.15610 (9)	0.45239 (12)	0.09457 (9)	0.0223 (3)	
C18	0.15587 (9)	0.36996 (13)	0.05588 (9)	0.0232 (3)	
C19	0.15470 (9)	0.27065 (12)	0.01184 (9)	0.0219 (3)	
C20	0.12070 (9)	0.26396 (13)	−0.06716 (9)	0.0237 (4)	
H20	0.0977	0.3257	−0.0905	0.028*	
C21	0.11988 (9)	0.16932 (13)	−0.11210 (10)	0.0256 (4)	
C22	0.15239 (10)	0.07952 (13)	−0.07467 (10)	0.0300 (4)	
H22	0.1519	0.0136	−0.104	0.036*	
C23	0.18519 (9)	0.08346 (13)	0.00358 (10)	0.0281 (4)	
H23	0.2058	0.0204	0.0275	0.034*	
C24	0.18827 (9)	0.17957 (12)	0.04787 (9)	0.0230 (3)	
C25	0.22660 (9)	0.18904 (12)	0.12662 (10)	0.0235 (4)	
C26	0.25924 (9)	0.20612 (12)	0.19125 (10)	0.0240 (4)	
C27	0.29570 (9)	0.23181 (12)	0.26867 (9)	0.0228 (3)	
C28	0.29559 (9)	0.16136 (14)	0.33603 (10)	0.0289 (4)	
H28	0.2728	0.0932	0.3298	0.035*	
C29	0.32799 (9)	0.18895 (13)	0.41168 (10)	0.0286 (4)	
H29	0.3269	0.1394	0.4564	0.034*	
C30	0.36228 (9)	0.28784 (13)	0.42378 (9)	0.0238 (4)	
C31	0.36368 (9)	0.35806 (13)	0.35677 (9)	0.0225 (3)	
H31	0.387	0.4257	0.3636	0.027*	
C32	0.33159 (9)	0.33165 (12)	0.27927 (9)	0.0213 (3)	
C33	0.33649 (9)	0.40474 (12)	0.21094 (9)	0.0224 (3)	
C34	0.34108 (9)	0.46519 (12)	0.15295 (9)	0.0233 (4)	
C35	0.34333 (9)	0.53578 (12)	0.08726 (9)	0.0232 (4)	
C36	0.34260 (9)	0.59846 (12)	0.03031 (9)	0.0228 (3)	
C37	0.41549 (10)	0.71348 (14)	−0.25636 (10)	0.0301 (4)	
H37	0.3804	0.7324	−0.303	0.036*	
C38	0.48096 (10)	0.79092 (15)	−0.26042 (11)	0.0377 (5)	
H38A	0.4631	0.8642	−0.2528	0.057*	
H38B	0.5045	0.7847	−0.3148	0.057*	
H38C	0.5173	0.7735	−0.2163	0.057*	
C39	0.44060 (12)	0.59924 (15)	−0.27061 (12)	0.0442 (5)	
H39A	0.4774	0.5793	−0.2277	0.066*	
H39B	0.4628	0.5934	−0.3257	0.066*	
H39C	0.3977	0.5512	−0.2676	0.066*	
C40	0.10830 (9)	0.81729 (13)	0.44585 (9)	0.0261 (4)	
H40	0.1495	0.8419	0.4838	0.031*	
C41	0.08181 (10)	0.70983 (14)	0.47865 (10)	0.0315 (4)	
H41A	0.0394	0.685	0.4449	0.047*	
H41B	0.0668	0.7177	0.5367	0.047*	
H41C	0.1222	0.6576	0.4755	0.047*	
C42	0.04634 (10)	0.89944 (14)	0.45132 (10)	0.0308 (4)	
H42A	0.0642	0.9687	0.4315	0.046*	
H42B	0.0309	0.9062	0.5093	0.046*	
H42C	0.0039	0.8764	0.4167	0.046*	
C43	0.08507 (10)	0.16014 (14)	−0.19909 (10)	0.0317 (4)	
H43	0.1231	0.1266	−0.2354	0.038*	
C44	0.06418 (11)	0.26707 (15)	−0.23850 (10)	0.0359 (4)	
H44A	0.0244	0.3003	−0.2067	0.054*	
H44B	0.0471	0.2555	−0.296	0.054*	
H44C	0.1077	0.3142	−0.2381	0.054*	
C45	0.01798 (11)	0.08507 (15)	−0.19878 (11)	0.0392 (5)	
H45A	0.0326	0.0164	−0.1741	0.059*	
H45B	0.0001	0.0736	−0.256	0.059*	
H45C	−0.0217	0.1171	−0.166	0.059*	
C46	0.40087 (9)	0.31230 (14)	0.50654 (9)	0.0294 (4)	
H46	0.3752	0.27	0.5504	0.035*	
C47	0.39803 (11)	0.42839 (15)	0.53199 (11)	0.0393 (5)	
H47A	0.4255	0.4716	0.492	0.059*	
H47B	0.4205	0.4367	0.5876	0.059*	
H47C	0.3462	0.4522	0.5329	0.059*	
C48	0.48084 (12)	0.2742 (2)	0.50436 (13)	0.0581 (7)	
H48A	0.4818	0.1969	0.4948	0.087*	
H48B	0.5057	0.2906	0.5576	0.087*	
H48C	0.5066	0.3106	0.4592	0.087*	

### Atomic displacement parameters (Å^2^)

**Table:**

	*U*^11^	*U*^22^	*U*^33^	*U*^12^	*U*^13^	*U*^23^
C1	0.0265 (8)	0.0179 (8)	0.0181 (7)	−0.0032 (7)	−0.0015 (6)	0.0005 (6)
C2	0.0273 (9)	0.0204 (8)	0.0212 (8)	−0.0019 (7)	−0.0010 (6)	−0.0032 (6)
C3	0.0281 (9)	0.0263 (9)	0.0174 (8)	−0.0052 (7)	−0.0001 (6)	−0.0020 (6)
C4	0.0298 (9)	0.0244 (9)	0.0208 (8)	−0.0027 (7)	−0.0001 (7)	0.0048 (7)
C5	0.0274 (9)	0.0193 (8)	0.0251 (8)	0.0011 (7)	0.0005 (7)	0.0019 (6)
C6	0.0240 (8)	0.0198 (8)	0.0196 (8)	−0.0029 (7)	0.0001 (6)	−0.0008 (6)
C7	0.0268 (8)	0.0158 (8)	0.0238 (8)	−0.0017 (7)	−0.0011 (7)	0.0006 (6)
C8	0.0272 (8)	0.0154 (8)	0.0237 (8)	−0.0013 (7)	−0.0002 (7)	−0.0001 (6)
C9	0.0250 (8)	0.0194 (8)	0.0200 (8)	0.0021 (7)	0.0001 (6)	−0.0015 (6)
C10	0.0321 (9)	0.0175 (8)	0.0296 (9)	−0.0049 (7)	0.0045 (7)	−0.0040 (7)
C11	0.0329 (9)	0.0209 (9)	0.0269 (9)	−0.0021 (7)	0.0035 (7)	−0.0084 (7)
C12	0.0232 (8)	0.0218 (8)	0.0220 (8)	0.0043 (7)	0.0008 (6)	−0.0030 (6)
C13	0.0251 (8)	0.0170 (8)	0.0204 (8)	0.0016 (7)	0.0001 (6)	−0.0001 (6)
C14	0.0230 (8)	0.0162 (8)	0.0200 (7)	0.0024 (7)	−0.0019 (6)	−0.0026 (6)
C15	0.0280 (9)	0.0186 (8)	0.0182 (8)	0.0013 (7)	0.0019 (6)	0.0015 (6)
C16	0.0271 (9)	0.0190 (8)	0.0186 (8)	0.0000 (7)	−0.0001 (6)	0.0024 (6)
C17	0.0285 (9)	0.0185 (8)	0.0198 (8)	0.0002 (7)	−0.0009 (6)	−0.0003 (6)
C18	0.0266 (9)	0.0218 (8)	0.0212 (8)	0.0004 (7)	0.0004 (6)	0.0002 (7)
C19	0.0259 (8)	0.0179 (8)	0.0220 (8)	−0.0006 (7)	0.0025 (6)	−0.0043 (6)
C20	0.0274 (9)	0.0210 (8)	0.0228 (8)	0.0024 (7)	−0.0004 (7)	−0.0017 (6)
C21	0.0278 (9)	0.0244 (9)	0.0247 (8)	0.0020 (7)	−0.0013 (7)	−0.0062 (7)
C22	0.0384 (10)	0.0209 (9)	0.0306 (9)	0.0025 (8)	−0.0061 (8)	−0.0100 (7)
C23	0.0354 (10)	0.0175 (8)	0.0311 (9)	0.0039 (7)	−0.0051 (7)	−0.0039 (7)
C24	0.0263 (8)	0.0213 (8)	0.0214 (8)	−0.0015 (7)	−0.0004 (6)	−0.0019 (6)
C25	0.0300 (9)	0.0156 (8)	0.0251 (8)	−0.0001 (7)	0.0020 (7)	−0.0015 (6)
C26	0.0314 (9)	0.0163 (8)	0.0243 (8)	−0.0020 (7)	0.0015 (7)	0.0011 (6)
C27	0.0271 (9)	0.0211 (8)	0.0203 (8)	0.0009 (7)	0.0001 (6)	0.0004 (6)
C28	0.0365 (10)	0.0219 (9)	0.0283 (9)	−0.0067 (8)	−0.0015 (7)	0.0037 (7)
C29	0.0359 (10)	0.0275 (9)	0.0225 (8)	−0.0015 (8)	0.0010 (7)	0.0076 (7)
C30	0.0259 (8)	0.0255 (9)	0.0202 (8)	0.0054 (7)	0.0014 (6)	−0.0001 (7)
C31	0.0251 (8)	0.0192 (8)	0.0232 (8)	0.0006 (7)	0.0002 (6)	−0.0013 (6)
C32	0.0254 (8)	0.0191 (8)	0.0194 (8)	0.0030 (7)	0.0013 (6)	0.0002 (6)
C33	0.0269 (9)	0.0186 (8)	0.0215 (8)	−0.0006 (7)	−0.0021 (6)	−0.0030 (7)
C34	0.0289 (9)	0.0188 (8)	0.0221 (8)	−0.0002 (7)	−0.0011 (7)	−0.0025 (7)
C35	0.0292 (9)	0.0192 (8)	0.0211 (8)	−0.0002 (7)	0.0002 (7)	−0.0016 (6)
C36	0.0267 (9)	0.0196 (8)	0.0221 (8)	−0.0008 (7)	−0.0001 (7)	−0.0035 (7)
C37	0.0373 (10)	0.0342 (10)	0.0188 (8)	−0.0040 (8)	0.0036 (7)	−0.0022 (7)
C38	0.0444 (11)	0.0346 (11)	0.0346 (10)	−0.0041 (9)	0.0157 (8)	−0.0031 (8)
C39	0.0608 (13)	0.0367 (11)	0.0358 (10)	−0.0074 (10)	0.0218 (9)	−0.0126 (8)
C40	0.0319 (9)	0.0263 (9)	0.0203 (8)	0.0034 (8)	0.0017 (7)	−0.0054 (7)
C41	0.0420 (11)	0.0312 (10)	0.0216 (8)	0.0035 (8)	0.0060 (7)	0.0005 (7)
C42	0.0346 (10)	0.0300 (10)	0.0278 (9)	0.0053 (8)	0.0055 (7)	−0.0044 (7)
C43	0.0375 (10)	0.0329 (10)	0.0245 (9)	0.0040 (8)	−0.0054 (7)	−0.0093 (7)
C44	0.0410 (11)	0.0415 (11)	0.0251 (9)	0.0019 (9)	−0.0056 (8)	−0.0020 (8)
C45	0.0453 (11)	0.0364 (11)	0.0356 (10)	−0.0001 (9)	−0.0124 (9)	−0.0080 (8)
C46	0.0348 (10)	0.0362 (10)	0.0172 (8)	0.0052 (8)	−0.0012 (7)	−0.0009 (7)
C47	0.0482 (12)	0.0394 (11)	0.0299 (10)	−0.0024 (9)	−0.0119 (8)	−0.0046 (8)
C48	0.0526 (14)	0.0865 (18)	0.0347 (11)	0.0291 (13)	−0.0175 (10)	−0.0187 (11)

### Geometric parameters (Å, °)

**Table:**

C1—C2	1.403 (2)	C29—C30	1.394 (2)
C1—C6	1.409 (2)	C29—H29	0.95
C1—C36	1.434 (2)	C30—C31	1.390 (2)
C2—C3	1.391 (2)	C30—C46	1.520 (2)
C2—H2	0.95	C31—C32	1.403 (2)
C3—C4	1.394 (2)	C31—H31	0.95
C3—C37	1.521 (2)	C32—C33	1.432 (2)
C4—C5	1.380 (2)	C33—C34	1.204 (2)
C4—H4	0.95	C34—C35	1.377 (2)
C5—C6	1.398 (2)	C35—C36	1.205 (2)
C5—H5	0.95	C37—C39	1.517 (3)
C6—C7	1.436 (2)	C37—C38	1.528 (2)
C7—C8	1.204 (2)	C37—H37	1
C8—C9	1.433 (2)	C38—H38A	0.98
C9—C10	1.398 (2)	C38—H38B	0.98
C9—C14	1.408 (2)	C38—H38C	0.98
C10—C11	1.386 (2)	C39—H39A	0.98
C10—H10	0.95	C39—H39B	0.98
C11—C12	1.394 (2)	C39—H39C	0.98
C11—H11	0.95	C40—C42	1.520 (2)
C12—C13	1.388 (2)	C40—C41	1.523 (2)
C12—C40	1.521 (2)	C40—H40	1
C13—C14	1.399 (2)	C41—H41A	0.98
C13—H13	0.95	C41—H41B	0.98
C14—C15	1.429 (2)	C41—H41C	0.98
C15—C16	1.206 (2)	C42—H42A	0.98
C16—C17	1.370 (2)	C42—H42B	0.98
C17—C18	1.204 (2)	C42—H42C	0.98
C18—C19	1.430 (2)	C43—C44	1.524 (2)
C19—C20	1.402 (2)	C43—C45	1.530 (3)
C19—C24	1.409 (2)	C43—H43	1
C20—C21	1.386 (2)	C44—H44A	0.98
C20—H20	0.95	C44—H44B	0.98
C21—C22	1.398 (2)	C44—H44C	0.98
C21—C43	1.526 (2)	C45—H45A	0.98
C22—C23	1.381 (2)	C45—H45B	0.98
C22—H22	0.95	C45—H45C	0.98
C23—C24	1.398 (2)	C46—C47	1.510 (2)
C23—H23	0.95	C46—C48	1.517 (3)
C24—C25	1.436 (2)	C46—H46	1
C25—C26	1.203 (2)	C47—H47A	0.98
C26—C27	1.433 (2)	C47—H47B	0.98
C27—C28	1.396 (2)	C47—H47C	0.98
C27—C32	1.415 (2)	C48—H48A	0.98
C28—C29	1.383 (2)	C48—H48B	0.98
C28—H28	0.95	C48—H48C	0.98
			
C2—C1—C6	119.60 (13)	C31—C32—C33	120.00 (14)
C2—C1—C36	120.68 (14)	C27—C32—C33	120.34 (13)
C6—C1—C36	119.71 (13)	C34—C33—C32	179.17 (18)
C3—C2—C1	121.45 (15)	C33—C34—C35	177.59 (18)
C3—C2—H2	119.3	C36—C35—C34	177.59 (18)
C1—C2—H2	119.3	C35—C36—C1	177.89 (17)
C2—C3—C4	117.98 (14)	C39—C37—C3	114.04 (14)
C2—C3—C37	122.91 (15)	C39—C37—C38	111.02 (15)
C4—C3—C37	119.06 (14)	C3—C37—C38	109.82 (13)
C5—C4—C3	121.64 (14)	C39—C37—H37	107.2
C5—C4—H4	119.2	C3—C37—H37	107.2
C3—C4—H4	119.2	C38—C37—H37	107.2
C4—C5—C6	120.69 (15)	C37—C38—H38A	109.5
C4—C5—H5	119.7	C37—C38—H38B	109.5
C6—C5—H5	119.7	H38A—C38—H38B	109.5
C5—C6—C1	118.62 (14)	C37—C38—H38C	109.5
C5—C6—C7	121.42 (14)	H38A—C38—H38C	109.5
C1—C6—C7	119.95 (13)	H38B—C38—H38C	109.5
C8—C7—C6	176.25 (16)	C37—C39—H39A	109.5
C7—C8—C9	175.33 (17)	C37—C39—H39B	109.5
C10—C9—C14	118.40 (14)	H39A—C39—H39B	109.5
C10—C9—C8	121.83 (14)	C37—C39—H39C	109.5
C14—C9—C8	119.70 (14)	H39A—C39—H39C	109.5
C11—C10—C9	120.41 (15)	H39B—C39—H39C	109.5
C11—C10—H10	119.8	C42—C40—C12	110.63 (13)
C9—C10—H10	119.8	C42—C40—C41	110.00 (14)
C10—C11—C12	121.90 (14)	C12—C40—C41	114.63 (13)
C10—C11—H11	119	C42—C40—H40	107.1
C12—C11—H11	119	C12—C40—H40	107.1
C13—C12—C11	117.63 (14)	C41—C40—H40	107.1
C13—C12—C40	122.84 (14)	C40—C41—H41A	109.5
C11—C12—C40	119.53 (14)	C40—C41—H41B	109.5
C12—C13—C14	121.67 (14)	H41A—C41—H41B	109.5
C12—C13—H13	119.2	C40—C41—H41C	109.5
C14—C13—H13	119.2	H41A—C41—H41C	109.5
C13—C14—C9	119.93 (14)	H41B—C41—H41C	109.5
C13—C14—C15	119.39 (14)	C40—C42—H42A	109.5
C9—C14—C15	120.64 (13)	C40—C42—H42B	109.5
C16—C15—C14	178.34 (17)	H42A—C42—H42B	109.5
C15—C16—C17	178.73 (18)	C40—C42—H42C	109.5
C18—C17—C16	178.88 (18)	H42A—C42—H42C	109.5
C17—C18—C19	178.44 (17)	H42B—C42—H42C	109.5
C20—C19—C24	120.06 (14)	C44—C43—C21	114.14 (14)
C20—C19—C18	120.17 (14)	C44—C43—C45	110.55 (15)
C24—C19—C18	119.78 (13)	C21—C43—C45	110.78 (14)
C21—C20—C19	121.53 (15)	C44—C43—H43	107
C21—C20—H20	119.2	C21—C43—H43	107
C19—C20—H20	119.2	C45—C43—H43	107
C20—C21—C22	117.49 (14)	C43—C44—H44A	109.5
C20—C21—C43	122.80 (15)	C43—C44—H44B	109.5
C22—C21—C43	119.72 (14)	H44A—C44—H44B	109.5
C23—C22—C21	122.16 (15)	C43—C44—H44C	109.5
C23—C22—H22	118.9	H44A—C44—H44C	109.5
C21—C22—H22	118.9	H44B—C44—H44C	109.5
C22—C23—C24	120.46 (15)	C43—C45—H45A	109.5
C22—C23—H23	119.8	C43—C45—H45B	109.5
C24—C23—H23	119.8	H45A—C45—H45B	109.5
C23—C24—C19	118.25 (14)	C43—C45—H45C	109.5
C23—C24—C25	122.27 (15)	H45A—C45—H45C	109.5
C19—C24—C25	119.44 (14)	H45B—C45—H45C	109.5
C26—C25—C24	174.43 (17)	C47—C46—C48	110.17 (17)
C25—C26—C27	176.80 (17)	C47—C46—C30	114.42 (14)
C28—C27—C32	118.09 (14)	C48—C46—C30	109.62 (14)
C28—C27—C26	121.65 (15)	C47—C46—H46	107.5
C32—C27—C26	120.24 (14)	C48—C46—H46	107.5
C29—C28—C27	121.18 (15)	C30—C46—H46	107.5
C29—C28—H28	119.4	C46—C47—H47A	109.5
C27—C28—H28	119.4	C46—C47—H47B	109.5
C28—C29—C30	121.52 (15)	H47A—C47—H47B	109.5
C28—C29—H29	119.2	C46—C47—H47C	109.5
C30—C29—H29	119.2	H47A—C47—H47C	109.5
C31—C30—C29	117.81 (14)	H47B—C47—H47C	109.5
C31—C30—C46	122.41 (15)	C46—C48—H48A	109.5
C29—C30—C46	119.65 (14)	C46—C48—H48B	109.5
C30—C31—C32	121.73 (15)	H48A—C48—H48B	109.5
C30—C31—H31	119.1	C46—C48—H48C	109.5
C32—C31—H31	119.1	H48A—C48—H48C	109.5
C31—C32—C27	119.64 (14)	H48B—C48—H48C	109.5
			
C6—C1—C2—C3	0.4 (2)	C22—C23—C24—C25	−175.30 (16)
C36—C1—C2—C3	−178.54 (15)	C20—C19—C24—C23	−1.3 (2)
C1—C2—C3—C4	−1.7 (2)	C18—C19—C24—C23	179.13 (15)
C1—C2—C3—C37	175.55 (15)	C20—C19—C24—C25	176.40 (15)
C2—C3—C4—C5	1.8 (2)	C18—C19—C24—C25	−3.2 (2)
C37—C3—C4—C5	−175.55 (15)	C32—C27—C28—C29	1.5 (2)
C3—C4—C5—C6	−0.6 (2)	C26—C27—C28—C29	−176.98 (16)
C4—C5—C6—C1	−0.7 (2)	C27—C28—C29—C30	−0.2 (3)
C4—C5—C6—C7	−179.51 (15)	C28—C29—C30—C31	−0.7 (2)
C2—C1—C6—C5	0.8 (2)	C28—C29—C30—C46	−176.65 (15)
C36—C1—C6—C5	179.77 (14)	C29—C30—C31—C32	0.3 (2)
C2—C1—C6—C7	179.63 (14)	C46—C30—C31—C32	176.14 (14)
C36—C1—C6—C7	−1.4 (2)	C30—C31—C32—C27	1.0 (2)
C14—C9—C10—C11	2.2 (2)	C30—C31—C32—C33	−177.81 (15)
C8—C9—C10—C11	−174.65 (15)	C28—C27—C32—C31	−1.9 (2)
C9—C10—C11—C12	−1.1 (3)	C26—C27—C32—C31	176.64 (15)
C10—C11—C12—C13	−1.1 (2)	C28—C27—C32—C33	176.94 (15)
C10—C11—C12—C40	178.95 (15)	C26—C27—C32—C33	−4.5 (2)
C11—C12—C13—C14	2.1 (2)	C2—C3—C37—C39	14.4 (2)
C40—C12—C13—C14	−177.86 (14)	C4—C3—C37—C39	−168.35 (16)
C12—C13—C14—C9	−1.0 (2)	C2—C3—C37—C38	−110.89 (18)
C12—C13—C14—C15	177.02 (14)	C4—C3—C37—C38	66.3 (2)
C10—C9—C14—C13	−1.2 (2)	C13—C12—C40—C42	−115.35 (17)
C8—C9—C14—C13	175.78 (14)	C11—C12—C40—C42	64.6 (2)
C10—C9—C14—C15	−179.21 (14)	C13—C12—C40—C41	9.7 (2)
C8—C9—C14—C15	−2.3 (2)	C11—C12—C40—C41	−170.28 (15)
C24—C19—C20—C21	−0.8 (2)	C20—C21—C43—C44	10.6 (2)
C18—C19—C20—C21	178.76 (15)	C22—C21—C43—C44	−169.67 (16)
C19—C20—C21—C22	1.8 (2)	C20—C21—C43—C45	−114.94 (18)
C19—C20—C21—C43	−178.43 (15)	C22—C21—C43—C45	64.8 (2)
C20—C21—C22—C23	−0.8 (3)	C31—C30—C46—C47	36.4 (2)
C43—C21—C22—C23	179.47 (16)	C29—C30—C46—C47	−147.78 (16)
C21—C22—C23—C24	−1.3 (3)	C31—C30—C46—C48	−87.9 (2)
C22—C23—C24—C19	2.3 (2)	C29—C30—C46—C48	87.9 (2)
